# Effects of a Loosening Meditation Practice on Older Adults With Mild Cognitive Impairment: A Pilot Study

**DOI:** 10.7759/cureus.78789

**Published:** 2025-02-09

**Authors:** Yoshinori Kajimoto, Shinobu Tamura, Kosuke Kawamura, Masato Kashiba, Etsuko Kishida, Hiroko Asada, Akihiro Yamamoto, Hidefumi Ito, Daien Oshita, Masaya Hironishi

**Affiliations:** 1 Internal Medicine, Wakayama Medical University Kihoku Hospital, Katsuragi-cho, JPN; 2 Nursing, Kyoto Tachibana University, Kyoto, JPN; 3 Psychiatry, School of Health and Nursing Science, Wakayama Medical University, Wakayama, JPN; 4 Nursing, Wakayama Medical University Kihoku Hospital, Katsuragi-cho, JPN; 5 Internal Medicine, Asada Clinic, Osaka, JPN; 6 Nursing, School of Health and Nursing Science, Wakayama Medical University, Wakayama, JPN; 7 Neurology, Wakayama Medical University, Wakayama, JPN; 8 Clinical Meditation, Hida-Senkoji Temple, Hida, JPN

**Keywords:** cognitive function, cortisol, loosening meditation, mild cognitive impairment, psychological well-being

## Abstract

Background

Holistic medical practices, including meditation and mindfulness, are increasingly recognized for their benefits in mental health and stress reduction, with applications in clinical settings. However, their effects on older adults with mild cognitive impairment (MCI) remain underexplored. This study aimed to evaluate the impact of loosening meditation, a brief and accessible practice we developed, on this population.

Methods

Nine older adults with MCI, defined by Mini-Mental State Examination (MMSE) scores of 21-28 during the screening period, participated in this pilot study. The sessions of our loosening meditation were conducted weekly over four weeks. Cognitive function was assessed using the MMSE and the Revised Hasegawa’s Dementia Scale (HDS-R), while psychological well-being was evaluated with the 30-item General Health Questionnaire (GHQ-30) and the Japanese version of the University of Wales Institute of Science and Technology Mood Adjective Checklist (JUMACL). Serum levels of representative stress biomarkers were measured before and after the first meditation session.

Results

Participants had a mean age of 78.1 years (± 4.8 years) and a mean MMSE score of 25.1 points (± 2.5). These participants could complete all sessions safely. No significant differences were observed in MMSE, HDS-R, GHQ-30, or JUMACL (tension arousal) scores before and after the intervention. However, JUMACL (emotion arousal) scores significantly improved after the fourth session (pre-first, mean 25.3 ± 6.2; post-fourth, mean 28.7 ± 7.3; p = 0.004). Among these stress biomarkers, serum cortisol levels significantly decreased following the first session (before versus after, mean 10.8 ± 5.8 versus 7.4 ± 2.3 μg/dl; p = 0.04). Additionally, although not statistically significant, there was a trend towards a lower level of adrenaline after the first session (before versus after, mean 0.057 ± 0.026 versus 0.050 ± 0.027 ng/ml; p = 0.06).

Conclusions

Our loosening meditation was feasible, acceptable, and safe, and might enhance mood and reduce stress even in older adults with MCI. These preliminary findings suggest its potential as a supportive intervention for this population.

## Introduction

Mild cognitive impairment (MCI) is a transitional stage between normal aging-related cognition and mild dementia [1]. Commonly observed in older adults, MCI is marked by declines in cognitive, memory, and attention functions [1]. With the aging population, the prevalence of MCI is rapidly increasing, making it a significant public health concern globally [2]. While some individuals with MCI revert to normal cognitive function or remain stable [3], others may progress to dementia, including Alzheimer’s disease (AD) [4,5]. Currently, no drug treatments are available to cure AD or other prevalent forms of dementia. A comprehensive approach that integrates holistic medicine with existing pharmacological treatments is essential to support the activities of daily living and overall well-being of individuals with MCI.

Cholinesterase inhibitors, such as donepezil, galantamine, and rivastigmine, are the standard treatments for AD and can stabilize or slightly slow cognitive decline [6]. The US Food and Drug Administration recently approved aducanumab, lecanemab, and donanemab, which are humanized monoclonal antibodies designed to reduce β-amyloid protein aggregates and slow cognitive decline [6-10]. However, these therapeutic antibodies show smaller cognitive benefits compared to cholinesterase inhibitors and may have limited effectiveness in early-stage AD [11]. A systematic review and meta-analysis indicated that low-dose lithium is more effective than aducanumab in early AD [12]. Considering the treatment of diseases, cost-effectiveness is crucial. Approaches that are affordable, acceptable to older adults, and easy to implement are essential, particularly for managing large populations with MCI and dementia.

In recent years, the stress-reducing benefits of meditation and mindfulness have gained global recognition in clinical practice. Kabat-Zinn et al. adapted meditation techniques by removing religious elements, integrating them with Western medicine, and applying them to healthcare [13-15]. Numerous clinical studies on mindfulness-based stress reduction (MBSR) and mindfulness-based cognitive therapy (MBCT) have demonstrated improved outcomes for various physical and psychological conditions [16]. Notably, MBSR, which incorporates mindfulness meditation, has shown greater effectiveness in adults with cognitive decline [17,18]. This practice also holds promise for slowing the progression of cognitive impairment in older adults.

Mindfulness meditation is widely recognized not only as a stress-reduction method but also as a means of fostering personal growth [13-15]. However, its successful implementation requires consistent participation by patients and proper training of instructors, presenting challenges for older adults with MCI in Japanese clinical settings. Based on these considerations, a mindfulness meditation practice is needed that (1) is easy to implement with older adults; (2) has the potential to improve cognitive function, reduce anxiety, and enhance happiness; and (3) is simple and free of side effects. Practices like MBSR and MBCT may be difficult for elderly individuals and those with dementia to follow. Thus, simplified meditation techniques that are more accessible could help mitigate cognitive dysfunction associated with dementia, including AD.

In this pilot study, we developed a loosening meditation practice designed to be feasible, acceptable, and safe for older adults with MCI and further assessed its effects on cognitive function, psychological well-being, and serum stress markers. Such interventions could serve as a non-pharmacological strategy to delay cognitive decline, improve emotional well-being, and enhance quality of life. This study aims to provide evidence supporting the integration of simple meditation techniques into clinical practice.

## Materials and methods

Patients and study design

**Figure 1:**
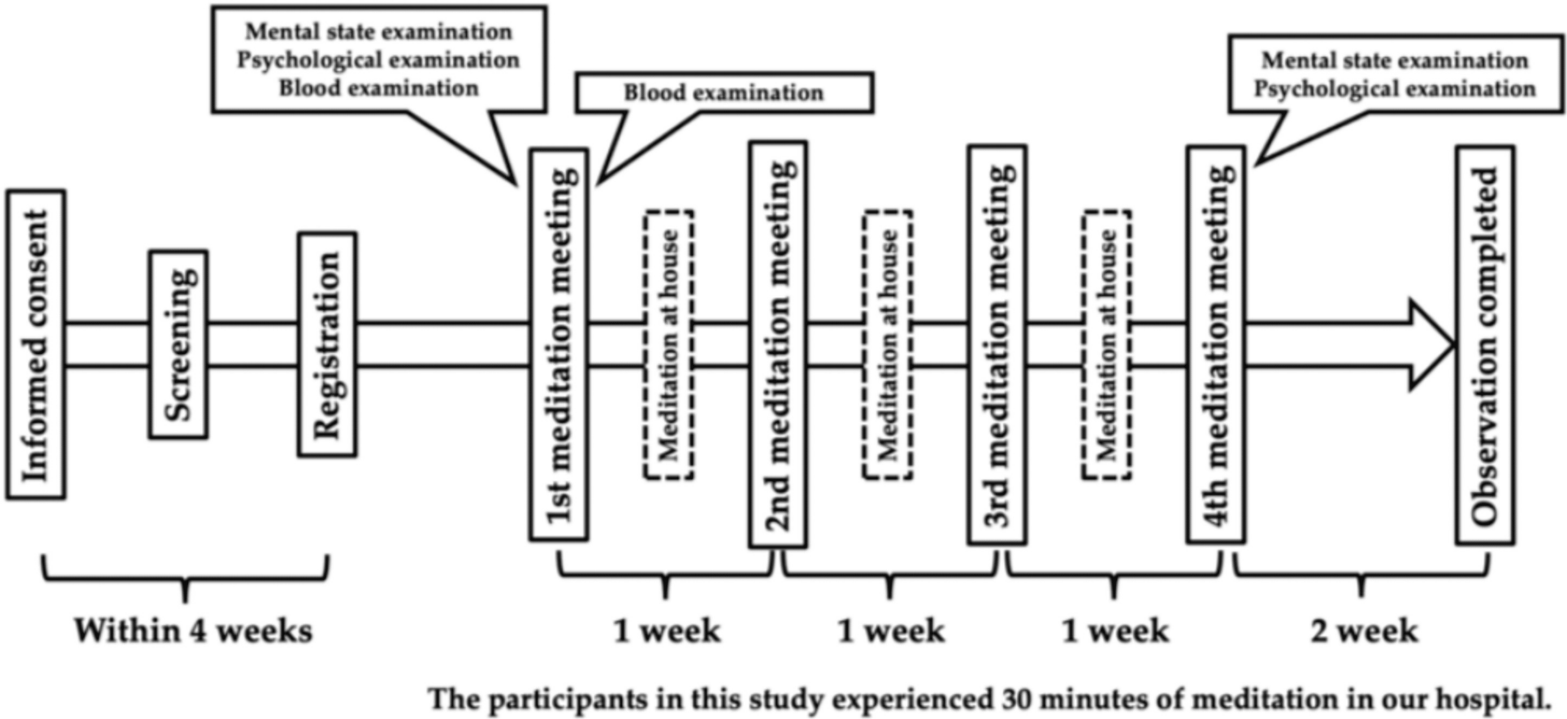
Study design

We designed the pilot study to assess the feasibility and acceptability of our loosening meditation to be planned in a larger-scale study in the future. The current study flowchart is presented in Figure 1. Patients were recruited from the Dementia Disease Medical Center at Wakayama Medical University Kihoku Hospital, Japan, between March 2022 and February 2024. Participants were eligible for inclusion if they met the following criteria: (i) aged over 20 years at the time of consent; (ii) able to attend meditation sessions conducted at the institute; (iii) diagnosed with mild neurocognitive disorder as defined by the Diagnostic and Statistical Manual of Mental Disorders, Fifth Edition; (iv) Mini-Mental State Examination (MMSE) scores between 21 and 24; and (v) capable of comprehending the written explanation of the study and providing written consent.

Participants were recruited through two methods: (1) direct invitations from their attending physicians during routine visits at the Dementia Disease Medical Center, and (2) self-referral by patients who noticed the 'Loosening Meditation Program' posters on hospital bulletin boards. This study adhered to the principles of the Declaration of Helsinki and was approved by the Ethical Review Board of Wakayama Medical University (approval no. 3404).

Intervention

The loosening meditation practice comprised five stages. First, the introduction (3 min), provides an overview of the session while participants sat comfortably with eyes gently closed. Second, deep breathing (2 min) that involves slow and deliberate breaths. Third, relaxation (7 min) that incorporates a guided body scan to release tension in specific areas. Fourth, meditation (15 min) that focuses on breathing while calmly redirecting wandering thoughts. And fifth, termination (5 min), i.e., gradually opening the eyes and sharing experiences with others. A summary of this practice is provided in Table 1.

**Table 1:** Specific practice of the loosening meditation

Themes	Time	Contents of the loosening meditation activity
Introduction	3 minutes	Overview of the practice
		Sit back in chair
		Close eyes lightly
Deep breath	2 minutes	Take a deep breath about 10 times, imagining your lung and expanding chest fully.
Relaxation	7 minutes	Perform a body scan and consciously release the tension in each area.
		Exhale and relax the tension while focusing on the below mentioned 10 areas in the same order:
		1) Around eyes and between eyebrows
		2) Cheeks and mouth area
		3) Back of head
		4) Shoulder and shoulder blade area
		5)Hands and fingers
		6) Chest and back
		7) Belly and lower back
		8) Gluteal region and inguinal area
		9) Thigh
		10) Lower leg and foot
		Find areas of tension and relax them again
Meditation	15 minutes	If thoughts wander, bring attention back to breathing.
		Distracting thoughts are not prohibited
Termination	5 minutes	Slowly open eyes
		Participants share their experiences with each other

The program consisted of weekly group sessions held at Wakayama Medical University Kihoku Hospital, with a total of four sessions. Participants were instructed to practice meditation at home at least twice a week. To support the practice at home, all participants were provided with a guided meditation DVD and a meditation instruction manual (see Appendix A).

Outcomes

The primary outcome was cognitive function, which was evaluated using the Japanese versions of the MMSE and the Revised Hasegawa’s Dementia Scale (HDS-R). Secondary outcomes included changes in emotional well-being using the 30-item General Health Questionnaire (GHQ-30) and the Japanese version of the University of Wales Institute of Science and Technology Mood Adjective Checklist (JUMACL) and stress biomarkers such as catecholamines (adrenaline, noradrenaline, and dopamine), adrenocortical steroids (dehydroepiandrosterone sulfate (DHEAS) and cortisol), and interleukin-6 (IL-6).

Clinical data collection

Patient medical records were retrospectively reviewed to obtain data on sex, age, and comorbidities. Cognitive and psychological assessments were conducted twice: before the first meditation session and after the fourth session. Cognitive and psychological assessments used in this study included the following measures: MMSE (score range 0-30), HDS-R (score range 0-30), GHQ30 (score range 0-30), and the JUMACL, which evaluated two dimensions: tension arousal (score range 10-40) and emotion arousal (score range 10-40). Stress biomarkers were analyzed using blood samples collected immediately before and after the first meditation session. These samples were sent directly to an outsourcing clinical laboratory (BML Inc., Saitama, JPN) for analysis. Serum catecholamines were measured using high-performance liquid chromatography. The range of normal values was less than 0.10 ng/mL for adrenaline, 0.10-0.50 ng/mL for noradrenaline, and less than 0.03 ng/mL for dopamine, respectively. Serum adrenocortical steroids were quantified using chemiluminescent immunoassay. The reference range of DHEAS was 50-2,530 ng/ml for men and 70-1,770 μg/dL for women aged ≥ 71 years, respectively. The normal reference range in the morning was 4.5-21.1 μg/dL for cortisol. Serum IL-6 was quantified using electrochemiluminescence immunoassay, and the normal range was less than 7.0 pg/mL.

Statistical analysis

Statistical analyses were conducted using GraphPad Prism 10 (GraphPad Software Inc., San Diego, CA, USA). Between-group comparisons for nonparametric continuous variables were performed using the Mann-Whitney U test. A p-value of <0.05 was considered statistically significant for all analyses.

## Results

This pilot study enrolled nine older adults with MCI, including four males. All participants provided informed consent independently. The clinical diagnoses included AD-MCI in four patients, Parkinson’s disease-MCI in three patients, vascular MCI in one patient, and multiple system atrophy-MCI in one patient [1,19-21]. At registration, the mean age was 78.1 ± 4.8 years, and the mean MMSE score was 25.1 ± 2.5 points. The baseline characteristics of the participants are summarized in Table 2.

**Table 2:** Clinical characteristics of nine older adults with mild cognitive impairment PD: Parkinson’s disease; AD: Alzheimer’s disease; MCI: Mild cognitive impairment; MSA: Multiple system atrophy; MMSE: Mini-Mental State Examination *MMSE points were evaluated during the screening period

Case	Age	Sex	MMSE*	Clinical diagnosis	Comorbidities
1	73	Male	28	PD-MCI	Restless legs syndrome
2	85	Female	21	AD-MCI	Hypertension
3	76	Male	23	Vascular MCI	Multiple cerebral infarctions, benign prostatic hyperplasia
4	74	Female	23	AD-MCI	None
5	83	Female	27	AD-MCI	Hypertension, gastric ulcer, osteoporosis
6	81	Female	24	AD-MCI	Gastric ulcer, gallstones, colorectal adenomas
7	83	Male	26	PD-MCI	Atrial fibrillation, angina pectoris
8	75	Male	28	PD-MCI	Hypertension, acute myocardial infarction, mesencephalic cavernous hemangioma
9	73	Female	26	MSA-MCI	Hypertension, dyslipidemia

**Figure 2:**
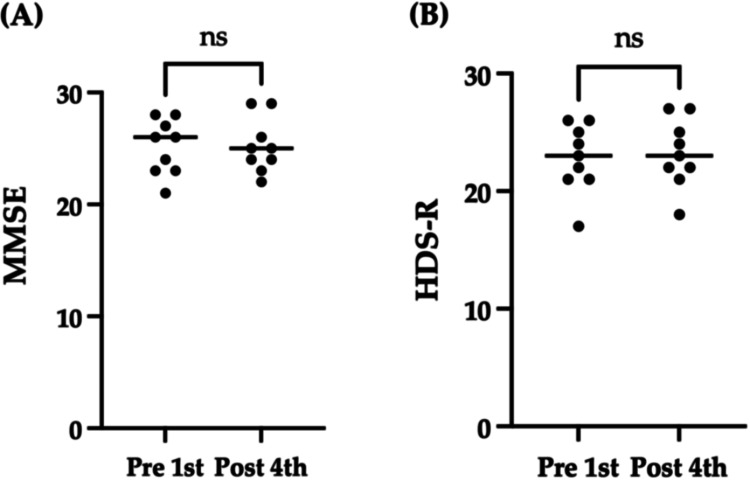
Comparison of cognitive function tests, namely the MMSE (A) and the HDS-R (B), before the first meditation session (pre-first) and after the fourth meditation session (post-fourth). Circles represent participants, and the black line indicates the medians. MMSE: Mini-Mental State Examination; HDS-R: Revised Hasegawa’s Dementia Scale; ns: Not significant

Cognitive function was assessed using the MMSE and HDS-R before the first session (pre-first) and after the fourth session (post-fourth) of the loosening meditation practice. The MMSE score showed no significant improvement from pre-first to post-fourth (pre-first, mean 25.1 ± 2.5; post-fourth, mean 25.2 ± 2.4; p = 0.98) (Figure 2A). Similarly, there was no clinically meaningful increase in the HDS-R score after the fourth session (pre-first, mean 22.8 ± 2.9; post-fourth, mean 23.2 ± 2.9; p = 0.70) (Figure 2B).

**Figure 3:**
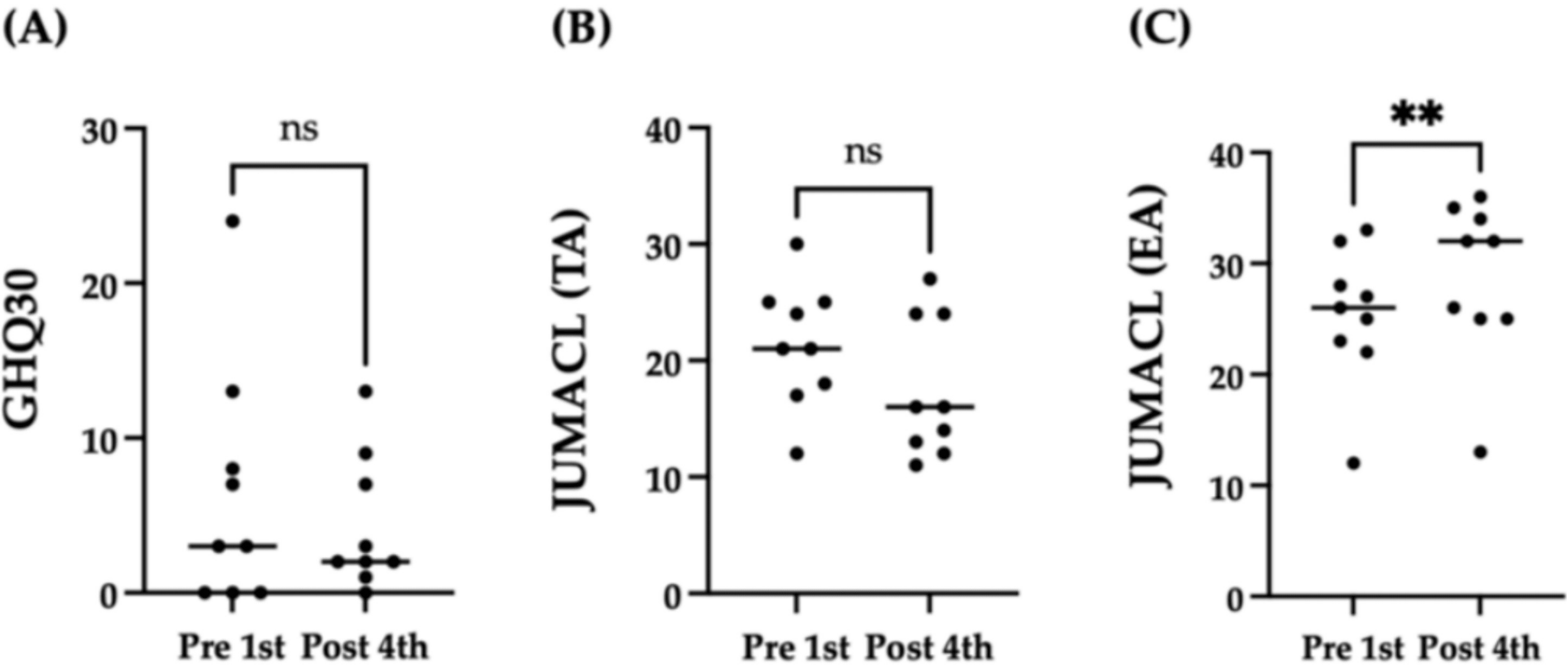
Comparison of the GHQ-30 (A) and the JUMACL (B, C) psychological tests, at pre-first and post-fourth. GHQ-30: 30-item General Health Questionnaire; JUMACL: Japanese version of the University of Wales Institute of Science and Technology Mood Adjective Checklist; ns: Not significant; TA: Tension arousal; EA: Emotion arousal The JUMACL was divided into two subscales, i.e., (B) TA, and (C) EA. Circles represent participants, and the black line indicates the medians. **p < 0.01

To assess changes in general mental well-being, the GHQ-30 and JUMACL (emotion and tension arousal subscales) were used as subjective measures at the pre-first and post-fourth sessions. A comparison of the results before and after the sessions showed no significant change in the GHQ-30 score (pre-first, mean 6.4 ± 7.9; post-fourth, mean 4.3 ± 4.4; p = 0.25) (Figure 3A). Similarly, no significant change was observed in the tension arousal subscale of the JUMACL (pre-first, mean 21.4 ± 5.3; post-fourth, mean 17.4 ± 6.0 points; p = 0.20) (Figure 3B). However, a statistically significant increase was observed in the emotion arousal subscale of the JUMACL from the pre-first to the post-fourth session (pre-first: mean 25.3 ± 6.2; post-fourth: mean 28.7 ± 7.3; p = 0.004).

**Figure 4:**
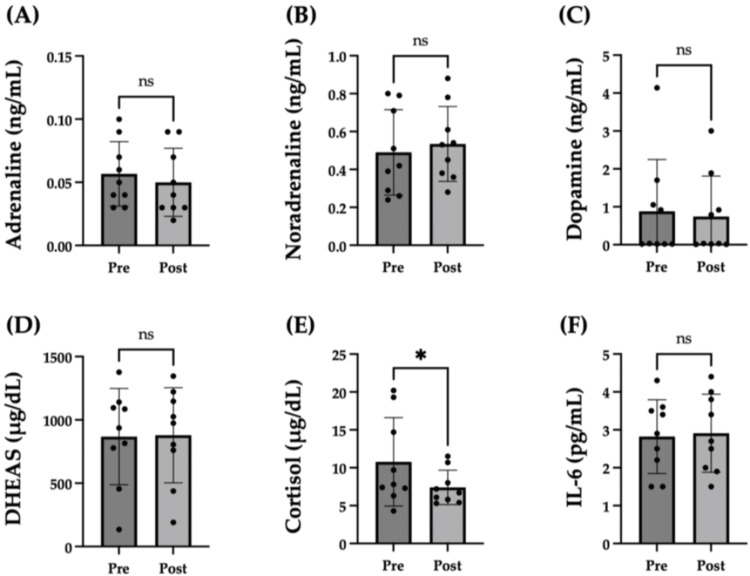
Comparison of representative serum stress biomarkers adrenaline (A), noradrenaline (B), dopamine (C), DHEAS (D), cortisol (E), and IL-6 (F) before and after the first meditation session DHEAS: Dehydroepiandrosterone sulfate; ns: Not significant Circles represent participants, and the top of the bar indicates the medians. *p < 0.05

Serum levels of catecholamines (adrenaline, noradrenaline, and dopamine), adrenocortical steroids (DHEAS and cortisol), and IL-6 were measured before and after the first session as objective indicators of stress. The noradrenaline level showed no significant change (before vs. after: mean 0.49 ± 0.22 vs. 0.53 ± 0.20 ng/ml; p = 0.34). Although the adrenaline level tended to decrease after the first session, it did not reach statistical significance (before vs. after: mean 0.057 ± 0.026 vs. 0.050 ± 0.027 ng/ml; p = 0.06) as shown in Figure 4A and Figure 4B. No significant change was observed in the levels of dopamine (before vs. after: mean 0.88 ± 1.37 vs. 0.74 ± 1.06 ng/ml; p = 0.44) and DHEAS (before vs. after: mean 869 ± 380 vs. 879 ± 375 μg/dl; p = 0.73) as depicted in Figure 4C and Figure 4D. However, the meditation practice significantly decreased the serum cortisol levels (before vs. after: mean 10.8 ± 5.8 vs. 7.4 ± 2.3 μg/dl; p = 0.04) (Figure 4E). No significant change was noted in the serum IL-6 levels before and after the session (before vs. after: mean 2.82 ± 0.97 vs. 2.91 ± 1.03 pg/ml; p = 0.38) (Figure 4F).

## Discussion

In this pilot study, nine participants completed all sessions of a loosening meditation. No significant change in cognitive function, including MMSE and HDS-R scores, was observed after conducting a loosening meditation. Regarding mental health and psychological distress, no significant changes were noted in the GHQ-30 and tension arousal subscale of JUMACL. However, the emotion arousal subscale of JUMACL showed a significant increase. Serum cortisol and adrenaline levels also tended to decrease after the first session. These results suggest that our short-term meditation practice effectively reduced stress in older adults with MCI.

The clinical effects of mindfulness and meditation have varied in previous studies. One report indicated that even short daily meditation could improve attention, memory, mood, and emotion regulation [22]. Moreover, mindfulness meditation has been shown to enhance psychological well-being by reducing stress, anxiety, and depression [23-25]. Interventional meditation can reduce emotional stress and improve cognitive function in older adults with worry symptoms and co-occurring cognitive dysfunction [26]. Moreover, meditation-based treatments have been found to reduce stress, slow cognitive decline, and improve the quality of life for patients with AD, MCI, and dementia [27]. This current study has shown that a loosening meditation could provide improvement in emotional stress for older adults with MCI; however, there was no significant change in cognitive function. Thus, further follow-up and evaluation are needed to clarify the cognitive improvement of the meditation.

Elevated cortisol levels are strongly associated with cognitive impairment, as often observed in patients with MCI, AD, and mental disorders [28,29]. Meditation has been clinically proven to improve serum stress biomarkers such as cortisol [30]. In this study, we found that our loosening meditation session significantly decreased serum cortisol in adults with MCI. Moreover, AD patients with MCI stage had recently shown high adrenaline concentration in cerebrospinal fluid (CSF), but not blood samples [31]. After the first session, a trend of decreasing serum adrenaline levels was observed, although without statistical significance, suggesting a potential decrease in CSF adrenaline. These findings suggest that measurement of serum cortisol and adrenaline may help predict the effectiveness of meditation sessions for older adults with MCI. Further investigations based on large samples and measurements after multiple sessions are required to clarify the precise association between cognitive impairment and serum stress biomarkers, particularly cortisol and adrenaline.

Previous studies have reported that mindfulness practice can improve cognitive function, anxiety, and depression in older adults with MCI [15]. However, a meta-analysis of randomized controlled trials on mindfulness-based interventions for dementia and MCI found no significant effects on any outcomes when compared to control conditions [32]. This meta-analysis cited several limitations, including the small number of studies, small sample sizes, and low quality of evidence. Therefore, further well-designed, large-scale trials of mindfulness and meditation are necessary to evaluate their effectiveness in treating cognitive impairment. This pilot study, in which all patients completed the sessions, demonstrates that our short-term meditation is feasible, acceptable, and implementable for older adults with MCI. From these findings, a novel intervention tailored to this population could be provided to promote and keep their motivation. Extended sessions over a longer period may lead to meaningful improvements in addressing cognitive decline as well as psychological stress. While cognitive function scales are often seen as key indicators of intervention outcomes in dementia, reducing stress and improving well-being in individuals with dementia should also be prioritized. The observed improvements in energy arousal and reductions in blood stress markers likely contributed to better mental well-being in these individuals, offering benefits that may not be captured by cognitive function scales.

At our institution, we provide psychological support to patients with COVID-19 through interviews with clinical psychologists who are Buddhist monks [33]. For many Japanese individuals, Buddhism is familiar and can promote mental and physical stability. It is often more sustainable, effective, and less complex than highly structured or complicated meditation methods. Building on this experience, we planned to offer a clinical meditation session (also known as a relaxing meditation meeting) for patients with MCI who could visit our institution. The primary goal was to provide psychological stability and improve daily living for individuals with cognitive impairment, anxiety, or stigma. This meditation practice was primarily developed by Buddhist priest Daien Oshita through his research [34]. In addition to his personal experience with Buddhist training and meditation, Oshita has studied and organized various meditation methods from different religious traditions.

According to Oshita, meditation can be categorized into four types: relaxing meditation, concentrating meditation, elevating meditation, and surrendering meditation. Relaxing meditation, which involves progressively relaxing the entire body through a body scan, serves as the foundational technique for all types of meditation. Concentrating meditation involves focusing thoughts and analyzing experiences and events. Elevating meditation seeks to fill the body and mind with energy. Surrendering meditation, with a somewhat spiritual aspect, aims to connect with the core of one’s existence, which could be understood as God, Buddha, or a greater universal energy. Among various methods, 'loosening meditation' focuses on relaxing both the mind and body, with the instructor guiding participants to relax each body part in stages, making it easier for individuals with MCI to practice.

In this study, none of the participants faced difficulty in practicing the meditation, refused to participate, or showed signs of confusion. The study provides preliminary yet promising results for mindfulness and meditation interventions benefiting individuals with dementia and their caregivers. Based on these findings, we have integrated the clinical meditation session into dementia care practices, including those for AD and vascular disease, at our hospital’s outpatient services.

Limitations

This study has several limitations. First, the small sample size as the study was conducted prospectively at our institute only. Second, blood tests were performed only before and after the first meditation session to reduce participant burden, so changes in stress biomarkers after subsequent sessions were unclear. Third, the meditation sessions were held once a week for four weeks to facilitate regular hospital visits; we are concerned that the stress-reduction effects may be offset by the repeated visits. Fourth, potential biases such as participant motivation to engage in meditation and variability in adherence to home practice must be considered. Future studies on larger cohorts and longer intervention durations should address these limitations.

## Conclusions

This pilot study showed that four weeks of loosening meditation sessions reduced psychological stress and stress biomarkers in elderly patients with MCI. Given its ease of implementation, this meditation method has the potential to benefit a wide range of individuals. Larger studies are needed to confirm its ability to slow dementia progression and support the daily functioning and mental health of patients with dementia.

## Figures and Tables

**Figure 5:**
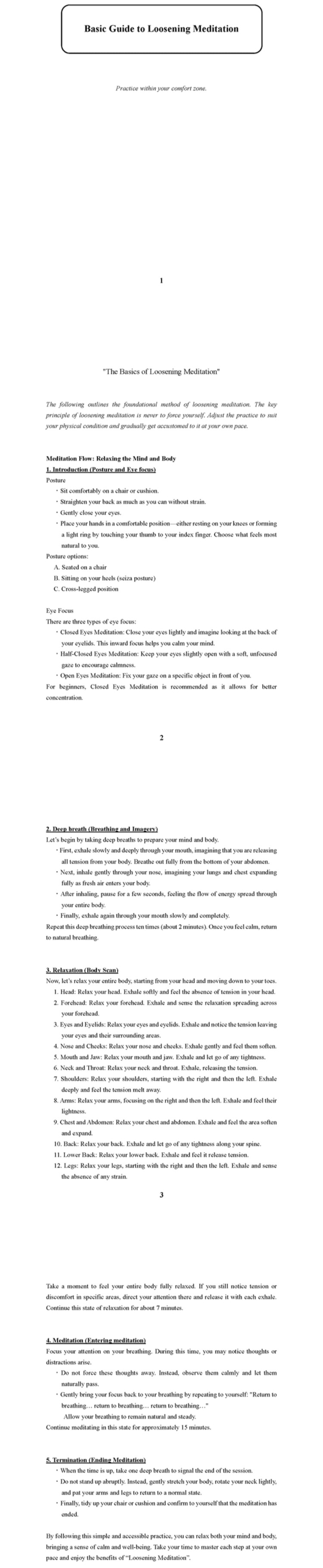
Meditation instruction manual

## Appendices

Appendix A

Figure 5 is a copy of the meditation instruction manual that was given to all participants to support their meditation practice at home. 
